# A Novel Dual Drug Approach That Combines Ivermectin and Dihydromyricetin (DHM) to Reduce Alcohol Drinking and Preference in Mice

**DOI:** 10.3390/molecules26061791

**Published:** 2021-03-22

**Authors:** Joshua Silva, Eileen Carry, Chen Xue, Jifeng Zhang, Jing Liang, Jacques Y. Roberge, Daryl L. Davies

**Affiliations:** 1 Titus Family Department of Clinical Pharmacy, University of Southern California School of Pharmacy, Los Angeles, CA 90089, USA; silvajos@usc.edu (J.S.); cxue4182@usc.edu (C.X.); jifengzh@usc.edu (J.Z.); jliang1@usc.edu (J.L.); 2 Molecular Design and Synthesis Group, Rutgers University Biomedical Research Innovation Core, Piscataway, NJ 08854, USA; eileen.carry@rutgers.edu (E.C.); jr1257@ored.rutgers.edu (J.Y.R.)

**Keywords:** dihydromyricetin, flavonoids, alcohol, alcohol use disorder, ivermectin

## Abstract

Alcohol use disorder (AUD) affects over 18 million people in the US. Unfortunately, pharmacotherapies available for AUD have limited clinical success and are under prescribed. Previously, we established that avermectin compounds (ivermectin [IVM] and moxidectin) reduce alcohol (ethanol/EtOH) consumption in mice, but these effects are limited by P-glycoprotein (Pgp/ABCB1) efflux. The current study tested the hypothesis that dihydromyricetin (DHM), a natural product suggested to inhibit Pgp, will enhance IVM potency as measured by changes in EtOH consumption. Using a within-subjects study design and two-bottle choice study, we tested the combination of DHM (10 mg/kg; i.p.) and IVM (0.5–2.5 mg/kg; i.p.) on EtOH intake and preference in male and female C57BL/6J mice. We also conducted molecular modeling studies of DHM with the nucleotide-binding domain of human Pgp that identified key binding residues associated with Pgp inhibition. We found that DHM increased the potency of IVM in reducing EtOH consumption, resulting in significant effects at the 1.0 mg/kg dose. This combination supports our hypothesis that inhibiting Pgp improves the potency of IVM in reducing EtOH consumption. Collectively, we demonstrate the feasibility of this novel combinatorial approach in reducing EtOH consumption and illustrate the utility of DHM in a novel combinatorial approach.

## 1. Introduction

Alcohol use disorder (AUD), characterized as a problematic pattern of alcohol use, affects over 18 million people and costs upwards of $250 billion annually in the United States [1,2]. Annual alcohol-associated deaths in the United States between 2011 and 2015 were over 93,000, with 54.7% of those deaths caused by chronic conditions and 45.2% by acute conditions [3]. Currently, there are three medications approved by the US Food and Drug Administration (FDA) to treat AUD: disulfiram, naltrexone, and acamprosate. Notably, patients with AUD often seek counseling to improve alcohol abstinence due to the lack of clinical success with pharmacological treatment [4]. The limited clinical success of available AUD therapeutics contributes to a low prescription rate of 9%, with medications only being prescribed to those who are likely to benefit from them, provided that clinically meaningful effects are observed [5]. This lack of effective therapeutics illustrates the necessity for identifying novel targets or innovative methods for the development of useful medications for the treatment of alcohol dependence and the consequential damage associated with alcohol abuse. 

Beyond improving the patient’s likelihood of success of alcohol abstinence, therapeutics are necessary to improve patient outcomes by reducing the risks of alcohol-related organ damage (AROD). With a lack of effective therapies for AUD, the persistent alcohol (ethanol/EtOH) abuse will exacerbate damage to the organs of the body, with substantial damage concentrated in the liver [6]. Notably, patients suffering from AUD also have a significant risk of developing alcoholic liver disease (ALD) in that the liver is the primary organ for metabolizing EtOH. Accordingly, the high mortality rate of ALD is related to the high probability of a return to drinking, with only 30–40% of newly diagnosed ALD patients remaining abstinent for one year [7]. This is not surprising when considering the low success rate of AUD treatment, with relapse being a common occurrence on the path to recovery [8,9]. Thus, a therapeutic strategy that can reduce the severity of AUD symptoms, meanwhile providing benefits to prevent AROD and ALD for the patient would be highly beneficial. 

A novel target that our laboratory has been working on for AUD is the P2X4 receptor [10,11,12,13,14,15]. P2X receptors are a family of cation-permeable ligand-gated ion channels activated by synaptically released extracellular adenosine 5′-triphosphate (ATP). Our lab has found that ivermectin (IVM), an FDA-approved semi-synthetic macrocyclic lactone, acts as a positive allosteric modulator (PAM) of P2X4 that reduces/eliminates the inhibitory effects of EtOH on P2X4 receptors [16,17,18,19,20]. Importantly, we demonstrated that IVM significantly reduced acute and chronic EtOH consumption in female and male C57BL/6J mice. IVM was found to have no effect on sucrose intake when tested at doses ranging from 0.65 mg/kg to 10 mg/kg and was well tolerated [12,13,15]. Adding to the evidence for a role of P2X4 as a target for EtOH and IVM, we found that male P2X4 knock-out (KO) mice resulted in higher EtOH intake than their respective controls and that there was a 50% reduction in IVM efficacy on EtOH intake in the P2X4 KO mice [12]. Together, this evidence suggests that P2X4 receptors represent a novel target for AUD drug development and support the utility of IVM-mediated positive allosteric modulation of P2X4 receptors as a novel therapeutic for AUD.

Although the utility of IVM for the treatment of AUD is supported, the pharmacokinetic (PK) properties of this drug limit its bioavailability to the CNS relative to other avermectins. For example, we found that moxidectin (MOX), a related avermectin, also modulated P2X4 receptors and antagonized EtOH’s inhibitory effects on ATP activity in vitro. In addition, MOX significantly reduced EtOH consumption in male and female C57BL/6J mice [11,21]. The findings from this work suggest that MOX has a superior PK profile in comparison to IVM, likely due to differences in efflux from the blood-brain barrier (BBB) and higher lipophilicity [11,13,21,22,23]. Interestingly, studies incorporating-glycoprotein (Pgp/ABCB1) deficient mice resulted in improved IVM PK profile as compared to MOX, suggesting that Pgp efflux activity of IVM is more robust compared to MOX in the CNS [22]. Furthermore, the high expression and activity of Pgp in the BBB is understood to limit the retention of IVM in the brain [24,25,26,27]. In agreement with these reported outcomes, we recently found that a novel dual drug strategy that used tariquidar (TQ), a potent non-substrate inhibitor of Pgp, in combination with IVM, significantly increased the potency of IVM as demonstrated by a 5-fold dose reduction of IVM necessary to reduce EtOH consumption in male C57BL/6J mice [28]. This work suggested that the regulation of Pgp efflux can enhance the positive allosteric modulation of P2X4 receptors in the CNS and thus improve the potency of IVM for AUD therapeutic benefits. 

Building evidence suggests that the flavonol component of a traditional herbal medicine [dihydromyricetin (DHM)] can be used to provide hepatoprotection by reducing the damage of chronic EtOH consumption and other drug-induced liver injuries in rodent models [29,30,31]. In addition, recent studies have shown that DHM can enhance the bioavailability of xenobiotic substrates of Pgp, suggesting that DHM acts as a Pgp inhibitor alongside other mechanisms that modify xenobiotic PK profiles [32,33,34]. Although the mechanism(s) associated with DHM’s ability to inhibit Pgp are unclear, recent structure-activity relationship (SAR) studies of flavonols and Pgp activity indicate that structural components of DHM are consistent with potent non-competitive Pgp inhibition of ATP hydrolysis [35]. For example, the 3′-OH, 4′-OH, and 2,3-saturation of DHM are associated with enhanced non-competitive Pgp inhibition [33,36,37]. Supporting these findings, taxifolin, a flavonol with a similar structure to DHM, was found to inhibit Pgp ATPase at concentrations as low as 100 nM through interactions with the Pgp nucleotide-binding domain (NBD). These findings collectively suggest that DHM can act as a non-competitive Pgp ATPase inhibitor, although further clarification on the mechanism is necessary. Therefore, we hypothesize that DHM, a compound that we have found to have hepatoprotective benefits [31], can inhibit Pgp and set the stage for a novel combination therapy that has the potential to reduce the severity of both AUD and ALD. To begin to test this hypothesis, we utilized a dual drug delivery approach of IVM plus DHM to assess the outcome of this unique combinatorial strategy on EtOH consumption and preference in C57BL/6J mice. Furthermore, we utilized an in silico approach to identify key ligand residue interactions to help advance our understanding of the use of DHM as a novel Pgp inhibitor in this combinatorial approach. 

## 2. Results

### 2.1. DHM Combined with IVM Significantly Increases IVM Potency on EtOH Intake and EtOH Preference

#### 2.1.1. Male Group Baseline Values

Male baseline values for EtOH consumption (g/kg) were found to be 11.89 g/kg/24 h for group 1, 11.76 g/kg/24 h for group 2, 12.64 g/kg/24 h for group 3, and 11.88 g/kg/24 h for group 4. No differences were observed between male groups or time points for both EtOH consumption (Data S1A, Supplementary Materials) and 10E preference (Data S1B). Comparisons of the day-to-day variability suggested that all male EtOH consumption values were not significantly different from the average baseline values and that all values were within range. Furthermore, Data S2 illustrates all daily measurements and changes of EtOH consumption (Data S2A) and preference (Data S2B) in male mice post-drug administration. Supplementary Data S3A, S4A, and S5A illustrate the daily values of male water intake (mL/kg/24 h), body weight (B.W.; %), and daily food intake (g), respectively. All doses of IVM + DHM were well tolerated, and no adverse responses were observed in male mice throughout the duration of this study.

#### 2.1.2. EtOH Intake (g/kg) Averages Compared between Male Treatment Groups

**Figure 1:**
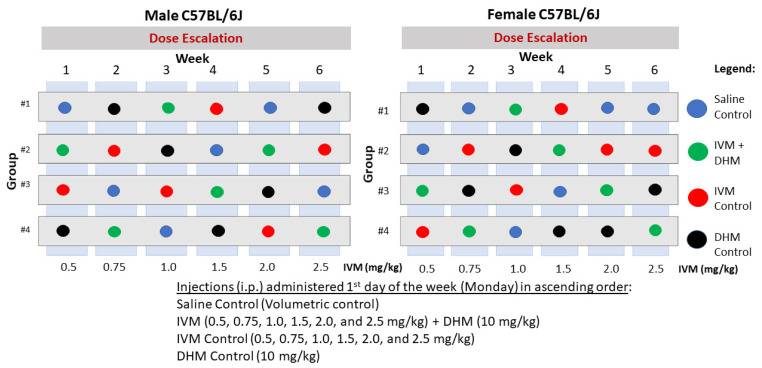
Randomized within-subjects drug treatment layout for behavioral analysis. Male and female C57BL/6J mice were separated into four cohorts and treated randomly each week with incremental doses of IVM as either (1) IVM dose control (red), (2) IVM + DHM (green), (3) Saline control (blue), and (4) DHM control (black).

**Figure 2:**
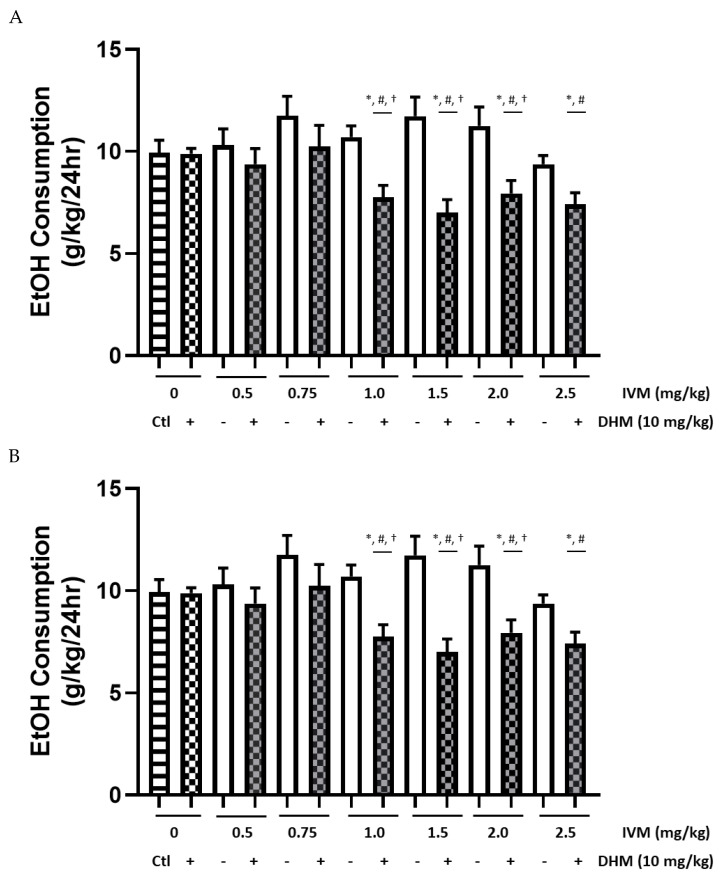
DHM (10 mg/kg) combined with IVM reduces the dosing necessary to significantly decrease EtOH consumption and 10E preference in male C57BL/6J mice over a period of 24 h. (**A**) IVM (1.0–2.5 mg/kg) combined with DHM (10 mg/kg) significantly reduced EtOH intake relative to saline treatment (Ctl), with 1.0–2.0 mg/kg IVM and DHM showing significant effects compared to IVM doses alone. (**B**) IVM (1.0–2.0 mg/kg) combined with DHM significantly reduces 10E preference in comparison to IVM controls. IVM (1.0–2.0 mg/kg) and DHM (10 mg/kg) significantly reduced 10E preference relative to saline values. Ctl = saline; DHM = dihydromyricetin; IVM = ivermectin. * *p* < 0.05 vs. Ctl values, † *p* < 0.05 vs. corresponding IVM dose control, and # *p* < 0.05 vs. DHM control; *n* = 48/group for DHM and saline groups; *n* = 8/group for IVM and IVM + DHM groups. All values are shown as averages ± SEM. 2-way ANOVA followed by Bonferroni’s multiple comparisons.

Having identified significant differences in the daily EtOH consumption (Data S2) using a randomized within-subjects design (Figure 1), we wanted to compare the changes in group averages of EtOH consumption with respect to treatment. To do so, we conducted a 2-way ANOVA that compared EtOH intake averages against the respective treatment groups over the 24 h period (Figure 2A), in which we identified a significant effect of treatment [F(13, 91) = 4.787, *p* < 0.0001]. Bonferroni’s multiple comparisons identified a significant reduction of EtOH intake post-administration of DHM + IVM [1.0–2.5 mg/kg] in comparison to the average of all saline (Ctl) post-treatment values collected over the 6-week study (*p < 0.05 for all comparisons). Similarly, a significant reduction of EtOH intake was identified post-administration when comparing the average of all post-treatment values of DHM controls compared to DHM + IVM [1.0–2.5 mg/kg] and 2.5 mg/kg IVM only (#*p* < 0.05 for all comparisons). The combinatorial drug strategy, when administered as DHM + IVM (1.0–2.0 mg/kg), showed significant differences when compared to their IVM controls (†*p* < 0.05 for all comparisons). However, no differences were observed between the combinatorial doses of IVM (0.5, 0.75, and 2.5 mg/kg) + DHM compared to the respective IVM control doses. Furthermore, when comparing the day-to-day variability throughout the male dose-escalation study, we found no significant differences in the average consumption values between all male mice. The highest concentration of EtOH consumed was in Group 1 at 12.25 g/kg/day (day 30; Data S1) and the lowest concentration was 8.875 g/kg/day in Group 4 (day 30; Data S1) following administration of DHM + IVM (2.5 mg/kg). Therefore, all EtOH consumption values were within range, with the lowest intake of EtOH following administration of the combined therapies.

#### 2.1.3. 10E Preference Averages Compared between Male Treatment Groups

We next wanted to identify differences in EtOH preferences after administration of the combinatorial therapy. A 2-way ANOVA of 10E preference values post drug administration in male groups (Figure 2B) identified a significant main effect of treatment [F(13, 91) = 9.076, *p* < 0.0001] on male EtOH preference averages. Bonferroni’s multiple comparison identified a significant reduction of 10E preference 24 h after administration of DHM + IVM [1.0–2.5 mg/kg] when compared to saline (Ctl) averages (Ctl; *p < 0.05 for all comparisons). Likewise, significant reductions of 10E preference was observed when comparing DHM control averages to the DHM + IVM [1.0–2.0 mg/kg] post-treatment averages (#*p* < 0.05 for all comparisons). Multiple comparisons of EtOH preference averages after administration of DHM + IVM compared to corresponding IVM controls identified a significant reduction in EtOH preference for DHM + IVM [1.0–2.0 mg/kg] when compared to the respective IVM control doses (†*p* < 0.05 for all comparisons).

#### 2.1.4. Female Group Baseline Values

Female baseline values were 13.4 g/kg/24 h for group 1, 15.2 g/kg/24 h for group 2, 14.4 g/kg/24 h for group 3, and 13.8 g/kg/24 h for group 4. No differences were observed between time or groups for both EtOH consumption (Data S1C) and 10E preference (Data S1D). Comparisons of the day-to-day variability suggested that all female EtOH consumption values were not significantly different from the average baseline values and that all values were within range. Furthermore, Data S3 illustrates all daily measurements of EtOH consumption (Data S3A) and preference (Data S3B) in female mice post-drug administration. Data S3B, S4B, and S5B illustrate no difference in female responses with treatment in water intake (mL/kg/24 h), body weight (B.W.) (%), and daily food intake (g), respectively. All doses of IVM + DHM were well tolerated, and no adverse responses were observed in female mice throughout the duration of this study.

#### 2.1.5. EtOH Intake (g/kg) Averages Compared between Female Treatment Groups 

**Figure 3:**
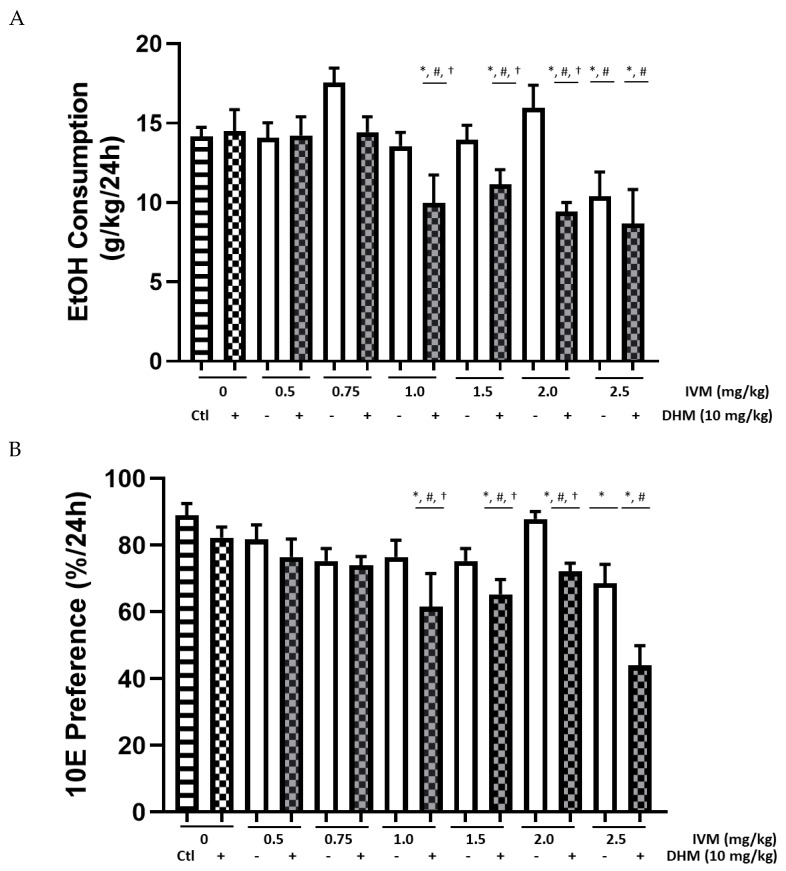
DHM (10 mg/kg) combined with IVM significantly reduces the dosing for EtOH consumption and 10E preference in female C57BL/6J mice over a period of 24 h. (**A**) IVM (1.0–2.5 mg/kg) combined with DHM (10 mg/kg) significantly reduced EtOH intake relative to saline treatment (Ctl), with 1.0–2.0 mg/kg IVM and DHM showing significant effects compared to IVM doses alone. (**B**) IVM (1.0–2.0 mg/kg) combined with DHM significantly reduces 10E preference in comparison to IVM controls. IVM (1.0–2.0 mg/kg) and DHM (10 mg/kg) significantly reduced 10E preference relative to saline values. Ctl = saline; DHM = dihydromyricetin; IVM = ivermectin. * *p* < 0.05 vs. Ctl values, † *p* < 0.05 vs. corresponding IVM dose control, and # *p* < 0.05 vs. DHM control; *n* = 48/group for DHM and saline groups; *n* = 8/group for IVM and IVM + DHM groups. All values are shown as averages ± SEM. 2-way ANOVA followed by Bonferroni’s multiple comparisons.

We next wanted to compare the changes in group averages of EtOH consumption after treatment in female C57BL/6J mice. Using a 2-way ANOVA, we found a significant effect of treatment [F(13, 91) = 4.927, *p* < 0.0001] (Figure 3A) on EtOH intake values in female mice. Bonferroni’s multiple comparison tests determined a significant reduction of EtOH intake after administration of DHM + IVM [1.0–2.5 mg/kg] and 2.5 mg/kg IVM in comparison to all saline (Ctl) controls (Ctl; *p < 0.05 for all comparisons). Similarly, a significant reduction of EtOH intake was observed when comparing the post-treatment averages of DHM controls against DHM + IVM [1.0–2.5 mg/kg] and 2.5 mg/kg IVM (#*p* < 0.05 for all comparisons). Bonferroni’s multiple comparisons of DHM + IVM compared to the corresponding IVM controls identified significant reductions of EtOH consumption with DHM + IVM [1.0–2.0 mg/kg] in comparison to the respective IVM controls (†*p* < 0.05 for all comparisons). When comparing the average EtOH intake values, no significant differences were observed between 2.5 mg/kg IVM alone vs. DHM + 2.5 mg/kg IVM. When comparing the day-to-day variability throughout the female dose-escalation study, we found no significant differences in the average values of all female mice, and the highest concentration of EtOH consumed was in Group 3 at 17.27 g/kg/day (day 7; Data S2), and the lowest concentration was 8.7 g/kg/day in Group 4 (day 27; Data S2) following administration of DHM + IVM (2.5 mg/kg). Therefore, all EtOH consumption values were within range, with the lowest intake of EtOH following administration of the combined therapies and correlating with our findings in male mice.

#### 2.1.6. 10E Preference Averages Compared between Female Treatment Groups

We next conducted a 2-way ANOVA to compare all 10E preference measurements against DHM and saline controls, as well as the respective IVM dose. We identified a significant main effect of treatment on female EtOH intake averages [F(13, 91) = 5.469, *p* < 0.0001] (Figure 3B). Bonferroni’s multiple comparison identified a significant reduction of 10E preference 24 h after administration of DHM + IVM [1.0–2.5 mg/kg] and 2.5 mg/kg IVM compared to saline (Ctl) controls (* *p* < 0.05). Similarly, a significant reduction of 10E preference in comparison to treatment with DHM was observed when compared against DHM + IVM [1.5–2.5 mg/kg] and 2.5 mg/kg IVM only (# *p* < 0.05). Multiple comparisons of DHM + IVM compared to corresponding IVM controls on 10E preference identified a significant reduction in EtOH consumption averages for DHM + IVM [1.0–2.0 mg/kg] in female mice (†*p* < 0.05 for all comparisons).

### 2.2. EtOH Consumption and Preference Averages Compared between Treatment Groups and Sex

#### 2.2.1. EtOH Consumption Averages (24 h Post-Injection) Compared between Treatment Groups and Sex

**Figure 4:**
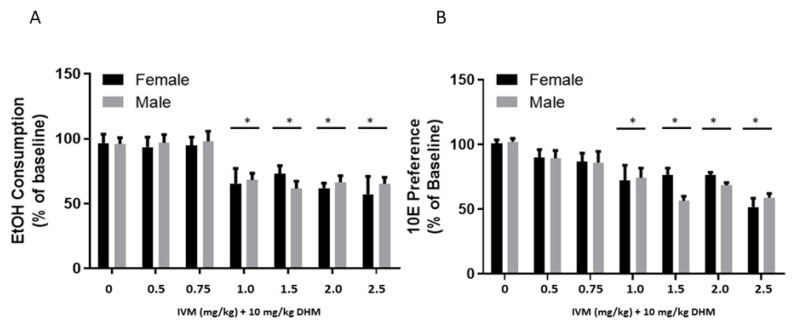
DHM (10 mg/kg) combined with IVM significantly reduces the dosing for EtOH consumption and 10E preference over 24 h in male and female C57BL/6J mice with no sex-specific differences. (**A**) DHM combined with IVM (1.0–2.5 mg/kg) significantly reduced EtOH consumption 24 h post-treatment with no sex-specific differences between normalized consumption values. Similarly, (**B**) DHM combined with IVM (1.0–2.5 mg/kg) significantly reduced 10E preference 24 h post-treatment with no sex-specific differences between normalized consumption values. IVM = ivermectin; DHM = dihydromyricetin; Baseline = day 0 values presented in Figure 1 and Figure 3 for males and females, respectively. * *p* < 0.05 vs. sex-matched baseline values (*n* = 48/group for DHM and saline controls; *n* = 8/group for IVM and IVM + DHM groups). All values are shown as averages ± SEM. 2-way ANOVA followed by Bonferroni’s multiple comparisons.

To identify sex-specific differences in the combinatorial potency, we utilized a repeated-measures 2-way ANOVA that examined sex and the effect of treatments on EtOH (g/kg/24 h) values followed by Bonferonni’s multiple comparisons (Figure 4A). We found a statistically significant main effect of treatment between EtOH consumption values [F(6, 42) = 9.419, *p* < 0.0001]. EtOH intake values (g/kg/24 h) were found to be significantly reduced after administration of DHM + IVM [1.0–2.5 mg/kg] in both male and female C57BL/6J mice when compared to saline (Ctl) controls (*p < 0.05 for all comparisons). Comparing male to female values of EtOH intake (g/kg/24 h) showed no difference between sexes, suggesting no sex-specific differences in EtOH consumption responses.

#### 2.2.2. 10E Preference Averages (24 h Post-Injection) Compared between Treatment Groups and Sex

To identify potential sex-specific differences in the therapeutic potency on 10E preference, we utilized a repeated-measures 2-way ANOVA that examined sex and the effect of treatments on 10E preference values followed by a Bonferonni’s multiple comparisons test (Figure 4B). Using a RM 2-way ANOVA, we found a statistically significant main effect of treatment on 10E preference [F(6, 42) = 12.95, *p* < 0.0001]. EtOH preference averages were found to be significantly reduced after administration of DHM + IVM [1.0–2.5 mg/kg] in both male and female mice when compared to saline (Ctl) controls (* *p* < 0.05 for all comparisons) with no differences between sexes.

### 2.3. In Silico Modeling Studies

**Figure 5:**
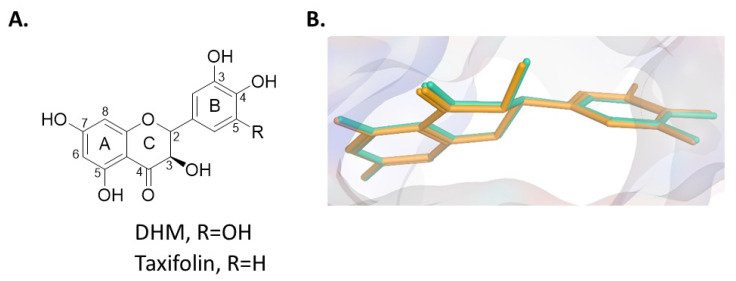
(**A**) Chemical structures of taxifolin (a potent Pgp ATPase inhibitor) and DHM, depicting variation only at the 5′ position of ring B. (**B**) Overlay of lowest binding energy conformations of taxifolin (green) and DHM (yellow) in NBD1 of human Pgp (PDB: 6C0V), depicting near-perfect overlap.

**Figure 6:**
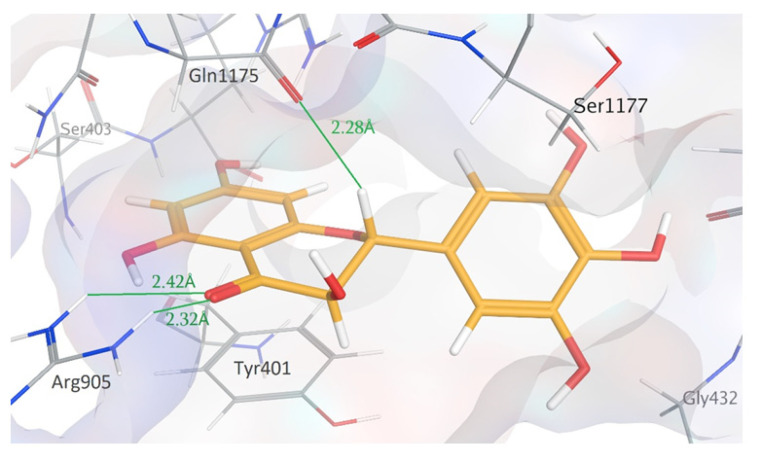
Lowest energy binding conformation of DHM (yellow) in NBD1 of human Pgp (PDB:6C0V). Atoms are displayed in the following colors: oxygen (red), nitrogen (blue), hydrogen (white), and carbon (grey, except DHM). Hydrogen bonds and distances are displayed in green. As depicted, in the lowest binding energy conformation, the 2-hydrogen and 3-carbonyl of DHM form hydrogen bond interactions with Gln1175 and Arg905 residues, respectively.

SAR studies of similar flavonoids support DHM to be a non-competitive Pgp inhibitor through interactions with the NBD. However, studies investigating non-competitive Pgp inhibition by DHM are lacking. Thus, to simulate non-competitive Pgp inhibition, in silico modeling studies were performed into the NBD1 of the Cryo-EM structure of human Pgp (PDB:6C0V) [38]. ATP, taxifolin, and DHM were successfully docked into the NBD1 binding site, with binding enthalpies of −16.1, −6.2, and −6.3, respectively (Figure 5, with lower binding enthalpies representing more favorable binding interactions. The comparable binding enthalpies between DHM and taxifolin suggest that DHM also acts as a non-competitive Pgp inhibitor. Further, docking results illustrate a near-perfect overlap between the lowest energy conformations of taxifolin and DHM (Figure 6), indicating a maintained binding conformation among DHM and taxifolin. Key residue-DHM interactions are displayed in Figure 6. Specifically, hydrogen bond interactions occur between the 4-carbonyl and 2-hydrogen of both DHM and taxifolin with Arg905 and Gln1175, respectively. These findings suggest that DHM acts as a non-competitive Pgp inhibitor through interactions at the NBD, maintaining key ligand-residue interactions of taxifolin.

## 3. Discussion

This is the first investigation demonstrating the utility of DHM as a regulator of Pgp activity that improves IVM potency on EtOH consumption in both male and female C57BL/6J mice. We found that the combination of DHM with IVM significantly increased IVM potency effects in reducing EtOH consumption and preference consistently in both sexes. These effects are likely due in part to DHM’s Pgp inhibiting activity resulting in increased CNS bioavailability of IVM. This finding is in agreement with our recent work, demonstrating that TQ improved IVM potency in male C57BL/6J mice [28]. In contrast, IVM alone at these effective doses did not show any significant effects on EtOH intake until it was administered at 2.5 mg/kg, further supporting our earlier findings of IVM (2.5–10 mg/kg) on EtOH consumption [13,28]. In support of DHM acting as a non-competitive Pgp inhibitor, molecular docking studies into NBD1 of Pgp revealed consistent ligand-residue interactions among DHM and taxifolin, a similar flavonoid and potent Pgp ATPases inhibitor. Combined, our findings support DHM as a promising Pgp inhibitor that can significantly enhance IVM potency in AUD models.

Molecular modeling studies have played a key role in interpreting and predicting non-competitive Pgp inhibition [39,40]. Notably, a direct correlation between Pgp NBD docking results of flavonoids and in vitro Pgp inhibition has been established [41], strongly supporting the validity of flavonoid docking into NBD of Pgp. With this in mind, we chose to utilize in silico modeling studies to illustrate critical DHM-Pgp interactions and mechanisms of Pgp inhibition. Upon docking into NBD1 of human Pgp, nearly identical binding conformations and enthalpies were observed between DHM and taxifolin, a potent Pgp ATPase inhibitor (Figure 6). Our results demonstrate that 2,3-saturation and 4-carbonyl of both DHM and taxifolin are involved in key ligand-residue interactions and support DHM as a Pgp ATPase inhibitor. The involvement of 2,3-saturation in binding pocket interactions correlates with SAR studies associating 2,3-saturation with enhanced potency of Pgp inhibition [35]. As SAR studies are reported for flavonoids similar to DHM, these data, coupled with modeling studies, could be utilized to guide the design of DHM analogs aimed at enhancing Pgp inhibition. For example, others have shown that *O*-methylation, prenylation of ring A at the 6 or 8 carbon, and addition of a 4′-*n*-octyl group results in enhanced potency Pgp inhibition. These findings are in line and support previous literature that has identified inhibition of Pgp with DHM and provide the first findings of key critical binding mechanisms of DHM to the NBD of human Pgp [32,33]. However, DHM modifications, which design towards Pgp inhibition/binding should be evaluated for differences in hepatoprotective effects observed with natural DHM [31,42,43,44,45]. Also, enhanced potency of Pgp inhibition could increase the risk of IVM neurotoxicity in the brain, in which IVM has been found to involve several mechanisms such as interactions with P2X4Rs, γ-aminobutyric acid type-A receptors (GABA_A_Rs), glycine receptors (GlyRs), and neuronal α7-nicotinic receptors (nAChRs) [46,47,48]. As such, future studies should also consider investigating the potential of enhancing the hepatoprotective effects of DHM while maintaining Pgp activity. These questions will be addressed in future investigations. Nonetheless, molecular modeling studies can provide a powerful tool to investigate the potential of DHM analogs for enhanced or maintained Pgp inhibition.

In support of our predictions of DHM enhancing the potency and safety of this combinatorial approach, we found that 10 mg/kg DHM in combination with IVM was well-tolerated up to the maximum dose that we tested (2.5 mg/kg). These observations of IVM + DHM being well tolerated at the maximum dose (2.5 mg/kg IVM) suggest an improvement in the safety of this combination of Pgp inhibition compared to TQ administration’s neurotoxic effects (10 mg/kg) combined with IVM at 2.5 mg/kg [28]. This improvement in drug tolerability is likely related to the hydrophilic properties of DHM that result in its rapid clearance, compared to potent longer-lasting inhibitors that block the conformation change of Pgp, such as TQ [28,49]. Importantly, we found that the combination of DHM with IVM between the doses of 1.0–2.0 mg/kg enhanced IVM’s potency to reduce EtOH intake in both male and female C57BL/6J mice, with no sex-specific differences. Interestingly, the combined dosing of IVM (above 1.0 mg/kg) with DHM did not provide any added effects in reducing EtOH intake. This lack of additional improvement by DHM + IVM may be due to a “ceiling effect”. Therefore, potential modifications of the DHM structure that benefit its PK profile may likely influence this “ceiling effect” and would be interesting to investigate for differences in combinatorial approaches. Of additional consideration is the potential impact that DHM may have on EtOH consumption. Previously, it has been reported that DHM can reduce EtOH voluntary intake in male SD rats when provided orally in tap water at 0.05 mg/mL and evaluated using a two-bottle choice paradigm [50]. In contrast, in the present study, we did not see a reduction in EtOH voluntary intake by DHM when administered at 10 mg/kg (i.p.). This could be due to the different types of animal models (SD rats vs. C57BL/6J mice) and metabolic differences resulting from differential metabolic enzyme expression and activity (e.g., cytochrome P450 [CYP] enzymes) [51], methodological differences in the delivery of DHM (i.p. vs. voluntary consumption) and/or frequency of DHM administration (once per week vs. daily administration). It has also been reported that the activity of DHM on GABA_A_R potentiation is critical to the anti-intoxication effects of DHM [50,52]. Therefore, it is of interest to explore the effects of DHM on saccharin and/or sucrose using the drinking model alongside investigations of changes when combined with IVM. Additionally, the enhanced metabolism of EtOH in the liver at the doses of 5 and 10 mg/kg DHM [31,42] is of interest when utilizing this combination and should be evaluated for changes in EtOH metabolism when administered as a combinatorial therapy. Continuing investigations utilizing other animal models and alcohol drinking assessments (e.g., drinking in the dark for evaluations of changes in “binge drinking”) will lead to a greater understanding of the benefits of DHM, either alone or in combination with IVM, as well as the effects of DHM on commonly tested tastants. Although these factors were not investigated or observed in this present study, our findings highlight the utility of DHM as a natural hepatoprotective molecule [31,42,43,53,54,55,56] that improves the potency of IVM in reducing voluntary consumption of EtOH. Furthermore, the development of this therapeutic combination supports our hypothesis that utilizing different types of Pgp inhibitors can improve the safety of this approach. However, future studies are necessary to better evaluate the observed ceiling effect of the combined therapy for potential differences relating to both alcohol intake and potential liver effects.

This novel combinatorial approach is not limited to the use of the investigated compounds (e.g., IVM as a P2X4 PAM). As mentioned earlier, MOX acts as a P2X4 PAM with a superior PK profile to IVM and significantly reduces EtOH drinking behavior [11,21]. Therefore, various combinatorial strategies utilizing different P2X4 PAMs and Pgp inhibitors need to be tested to identify combinations with the greatest therapeutic potential. In addition to identifying multiple P2X4 PAMs and/or Pgp inhibitors, future studies are necessary to expand the utility of these combinations in other animal models of AUD. For example, investigations focusing on the utility of this combinatorial approach in models of binge-like drinking (e.g., Drinking in the Dark [DID] Paradigm) and alcohol withdrawal would add therapeutic value to the current findings by expanding the therapeutic potential of this therapy beyond EtOH voluntary intake. Furthermore, the approach of using the DHM flavonoid in combination with IVM suggests that novel combinatorial strategies utilizing flavonoids, and other potential Pgp inhibitors, with known pharmacological responses can likely provide added systemic benefits against EtOH- or drug-mediated organ injury.

Previously, human studies found that IVM administered at a dose of 30 mg is well-tolerated in alcohol-dependent patients that were intravenously delivered an intoxicating dose of EtOH (0.8 g/dL; *n* = 11) [57]. Unfortunately, no clinical benefit in cue-induced cravings was observed from this small human subject investigation. However, it is important to note that this study was not designed nor powered to investigate IVM’s potential to reduce the patients’ level of alcohol consumption or change consumption habits. In addition, due to the time constraints of the clinical support staff, the effects of IVM were measured approximately 6 h after IVM administration. Our preclinical work suggests that IVM activity is not observed until approximately 9 h after administration [11,21]. Nonetheless, the lack of clinical benefit leads us to ask the question, is there a way to improve IVM potency for IVM activity in the CNS to support the use of IVM for AUD? As presented in our recent work [28] and the present study, the answer is yes. With this in mind, we predict that this dual drug strategy could be employed to improve the therapeutic potential of IVM in patients seeking pharmacotherapy for AUD. Notably, many patients undergoing AUD pharmacotherapy often revert to alcohol abuse, and thus patients need to be aware of potential risks of alcohol abuse with ongoing treatments [58]. The initial human safety study, as described above, lessens this concern as no significant adverse effects by IVM were noted compared to placebo [57].

Furthermore, consideration of the subsequent effects of alcohol abuse must be considered when developing therapies for patients suffering from AUD. Individuals that suffer from chronic alcohol abuse often develop systemic injuries, particularly the liver (e.g., ALD), where much of the damage is concentrated due to its primary role in EtOH metabolism [59,60,61,62,63,64,65]. To address this, our approach of combining a hepatoprotective agent with Pgp inhibiting activity (i.e., DHM) and IVM elicits reductions in EtOH consumption at lower and safer doses of IVM and can potentially provide benefits to the liver following EtOH metabolism [31,42,56]. Based on the novel aforementioned combinatorial findings that utilize a Pgp inhibitor with hepatoprotective benefits, we are currently investigating the potential utility of targeting both AUD and ALD via modifications of DHM hepatoprotection when combined with IVM. In support of this hypothesis, therapeutic benefits have been observed with IVM on nonalcoholic fatty liver disease, hyperglycemia, and lipidemia using in vitro and in vivo models, and these preclinical benefits are likely related to the activity of IVM on hepatic farsenoid X receptors (FXR) [66,67,68]. Based on these findings, ongoing research in our laboratory is focusing on the combination of DHM and IVM on the liver for potential changes in DHM mediated activation of AMP-activated protein kinase [AMPK] [31,42,45,69] and/or IVM activity on FXR when combined as a combinatorial therapy. Furthermore, this combinatorial therapy’s combined effects should also be investigated in other tissues, as Pgp inhibition or competition is likely to affect the PK profile in other Pgp expressing organs. 

As presented above, the current study outcomes relied on the use of a two-bottle choice assessment to evaluate EtOH consumption. This paradigm is commonly used as a behavioral model to assess murine EtOH consumption, in many instances similar to what is observed in “social drinking” [70]. Although a commonly used drinking paradigm, we should point out, as with the use of all models, there are limitations associated with environmental stressors. In the case of two-bottle choice studies, experimental conditions can influence murine self-administration. For example, unexpected noises or other irritations have been reported to affect self-administration in mouse models [70]. Furthermore, as this model relies on murine self-administration, there is expected variability with the collected values as all C57Bl/6J mice do not drink the same volumes. These variabilities likely depend on individual physiological variables (e.g., body weight) and a preference for one spout over the other due to the left- or right-handed preferences [71,72,73]. In recognizing these limitations, we utilize additional techniques that are well developed to address these assays’ concerns. To manage the potential right- or left-handed preference of bottles, we adjust both the EtOH and water bottles every other day to prevent selecting a solution over the other due to placement [28,70]. Furthermore, because of the variability of volumes that mice regularly ingest, we ensure a sufficient n value of mice, including a within-subjects design, to determine drug-dependent responses on EtOH intake and preference. We then utilize the ranges between all groups and individuals to ensure that the volume is within range (±10% variability) [28,70]. To ensure that these responses are consistent within and between models, these limitations will need to be considered as we begin exploring this novel combination in other alcohol drinking models.

In summary, we provide strong evidence for the utility of a combinatorial approach that implements a Pgp inhibitor and P2X4 PAM, IVM, to enhance the potency of IVM on EtOH intake behavior. Additionally, we are the first to report that using DHM in combination with IVM enhances the dosing effects and improved the safety of this approach when compared to the neurotoxic responses observed with the administration of TQ (10 mg/kg) and IVM (2.5 mg/kg) [28]. Supporting the mechanism of this promising synergistic effect, we report the first molecular modeling study to identify key DHM-NBD interactions that strongly support DHM to be a non-competitive Pgp inhibitor. Based on these novel findings of combining IVM with a Pgp inhibitor with hepatoprotective benefits, ongoing research will expand our analyses of this novel combinatorial strategy to elucidate the potential hepatoprotection of administering DHM in combination with IVM as an innovative therapy to target both AUD and ALD. Importantly, this study encourages the investigation of novel multi-targeted therapy combinations to benefit the treatment of AUD and other CNS disorders lacking therapeutic options, meanwhile providing pharmacological mechanisms that protect the patient from alcohol- or drug-induced organ damage.

## 4. Materials and Methods

### 4.1. Animals and Experimental Design

Thirty-two male and thirty-two female C57BL/6J mice (6-weeks of age) were purchased from Jackson Laboratories (Bar Harbor, ME, USA). C57BL/6J mice have been studied over many decades and are often utilized in alcohol studies due to their high alcohol intake and preference compared to other strains [74,75,76]. All mice were individually housed in light-, temperature-, and 40–60% humidity-controlled conditions with a 12 h light/dark cycle, and the vivarium was maintained at 22 °C. Experimental procedures were approved by the USC IACUC (protocol # 10977 approved 11 August 2020), and all studies were carried following the relevant regulations and guidelines.

### 4.2. Two-Bottle Choice EtOH Drinking Behavior 

The combination of IVM with DHM on EtOH consumption was evaluated using a two-bottle choice drinking paradigm providing free access to chow and two bottles (tap water and 10% ethanol [10E]) over the course of the 6-week study with the recognition that EtOH consumption varies between individuals due to the free access to alcohol, as previously described [13,14,21,28,77]. All mice were 8 weeks old at the start of the EtOH-drinking and dose-escalation studies. In all cases, injections were administered immediately prior to the period of 24 h access to 10E (10% EtOH *v*/*v* solution in tap water) versus tap water, so that the change in drinking over 24 h after drug administration was measured. Baseline EtOH consumption values were collected over a 10 day acclimation period in which all mice were administered two saline injections (i.p.) to control for the number of injections and volumes utilized within the study. These daily values (Data S1) were then averaged and used as the day 0 (baseline value) in Supplementary Data S2 and S3. Using a within-subjects design, randomized groups of mice (8 mice/group) received an i.p. injection with either DHM (10 mg/kg) followed by a second i.p. injection of a single dose of IVM (0.5–2.5 mg/kg) 15 min post-DHM. The remaining three groups were randomized into control groups receiving either saline, DHM (10 mg/kg), or the respective IVM dose control followed by a second saline i.p. injection (as a control for injections and volume). A within-subjects study design was utilized to 1) compare treatment effects between groups and simultaneous treatment, 2) compare effects against baseline EtOH consumption within the same population, 3) minimize the random noise between treatments, 4) identify potential sensitization effects on alcohol intake with treatments within the same populations, and 5) improve the humane use of laboratory animal models by minimizing the number of animals needed for treatment. Randomization of groups proceeded from week 1 (0.5 mg/kg IVM dose study)–week 6 (2.5 mg/kg IVM dose study) so that each group received at least one treatment of IVM + DHM and controls per week for effective comparisons between treatment groups and against baseline consumption values (Figure 1). All treatments (saline, DHM, IVM alone, and IVM + DHM) were administered once per week to the designated randomized group. Following the treatment period, all mice were then evaluated for changes in EtOH consumption and preference, followed by a 1-week wash-out period to ensure elimination of the treatment before the transition to the next dose of IVM randomized to another group. The dose of DHM utilized in this study was selected due to our previous work demonstrating reduced ALD in male C57BL/6J mice [31,42]. The IVM doses tested in this study were 0.5, 0.75, 1.0, 1.5, 2.0, and 2.5 mg/kg. IVM doses were administered ± DHM in ascending order in randomized groups after a 1-week wash-out period for all treatments, established as a return to baseline EtOH consumption, to ensure that the next administration of control(s) or combinatorial therapy would not confound the previous intervention. Dose escalation studies of EtOH intake (g/kg/24 h) and 10E preference continued until the final dose of IVM (2.5 mg/kg) that we have found to have significant effects on EtOH consumption when administered alone [13,14,28].

### 4.3. Statistical Analyses

GraphPad Prism (GraphPad Software, Inc., La Jolla, CA, USA) was used to conduct all statistical analyses. EtOH consumption and 10E preference values for all 4 randomized groups and treatments were analyzed using a repeated-measures (RM) 2-way ANOVA either as daily changes (Figure 1 [males] and Figure 3 [female]) or compiled as averages for comparisons of combinatorial administration vs. IVM alone between groups of the same sex (Figure 2 and Figure 4) using Bonferonni’s multiple comparisons test. Likewise, all data collected (*n* = 48 total/sex for saline and DHM; *n* = 8/sex for IVM controls and IVM + DHM) were compiled as averages of post-treatment values collected throughout the studies and compared to the post-treatment values of the opposite sex (Figure 3) using a Bonferonni’s multiple comparisons test. For data presented in Figure 2, Figure 3 and Figure 4, all values obtained from saline and DHM treatment groups collected throughout the study (*n* = 48) were averaged from all daily values over the 6 separate dose trials. These values were then compared against the averages of IVM alone or IVM + DHM values (*n* = 8/dose) to compare all dose treatments to the total average values using a RM 2-way ANOVA followed by Bonferroni’s multiple comparisons tests. Likewise, for data presented in Figure 4, all values obtained from saline and DHM controls (*n* = 48/sex) were averaged and compared against the respective IVM dose control (*n* = 8/sex) and IVM + DHM dose-matched treatments (*n* = 8/sex) for assessment of differences against control values within the same sex and treatment effects in the opposite sex. Statistical significance was set at *p* ≤ 0.05 for all studies.

### 4.4. In Silico Modeling Studies

To simulate non-competitive Pgp inhibition, in silico modeling studies were performed into the nucleotide-binding domain (NBD)1 (ATP binding site) of the Cryo-EM structure of human Pgp with bound ATP (PDB:6C0V) [38]. Molecular Operating Environment (MOE^®^, Chemical Computing Group Inc., Montreal, QC, Canada) software was utilized for modeling studies and energy minimization of all compounds. First, to ensure all ligands were in their lowest energy structural conformations, energy minimization was conducted on ATP, DHM, and taxifolin. Next, all ligands were docked with 1000 poses, using a triangular matcher method, and induced fit refinement. Of the docking poses, docking conformation of the lowest binding energy, signifying more favorable binding interactions, was selected for each ligand. To assess the accuracy of the docking simulation, we compared the lowest energy docking conformation of ATP with that of the Cryo-EM bound ATP. Complete structural overlap between docked and Cryo-EM ATP was observed, supporting the validity of our ligand docking results into NBD1 of human Pgp (PDB:6C0V).

## Data Availability

The data presented in this study are available on request from the corresponding author.

## Supplementary Materials

The following are available online. Data S1: Baseline values for male and female C57BL/6J mice groups, Data S2: IVM, combined with DHM, significantly reduced EtOH intake and 10E preference at lower doses in male C57BL/6J mice, Data S3: IVM, combined with DHM, significantly reduced EtOH intake and 10E preference at lower doses in female C57BL/6J mice, Data S4: Daily treatment effects on daily water intake (mL/kg/24 h), Data S5: Daily treatment effects on body-weight (B.W.; %)., Data S6: Daily treatment effects on daily food intake (g).

## Sample Availability

Samples of the compounds are not available from the authors.
