# Supplementary material for: A Novel Dual Drug Approach That Combines Ivermectin and Dihydromyricetin (DHM) to Reduce Alcohol Drinking and Preference in Mice
Source: Molecules. 2021 Mar 22;26(6):1791. doi: 10.3390/molecules26061791 (PMC8004700; doi:10.3390/molecules26061791)
Supplement: Supplementary file 1 [file molecules-26-01791-s001.pdf]

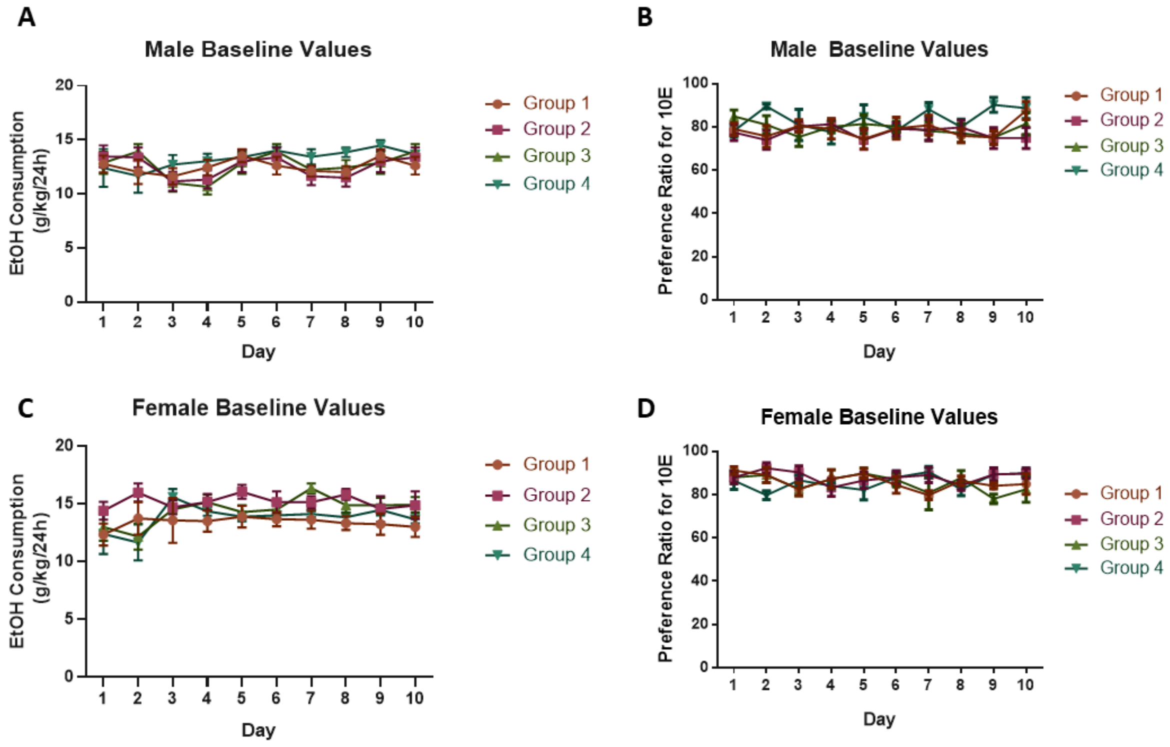

Data S1. Baseline values for male and female C57BL/6J mice groups. A) Daily EtOH consumption (g/kg/24-h) and B) daily 10E preference ratios for male groups (n=8/group). C) Daily EtOH consumption (g/kg/24-h) and D) daily 10E preference ratios for female groups (n=8/group). All values for each male or female group EtOH consumption/10E preference were averaged and used as Day 0 baseline values for each respective group, as represented in Data S2 and Data S3.

A

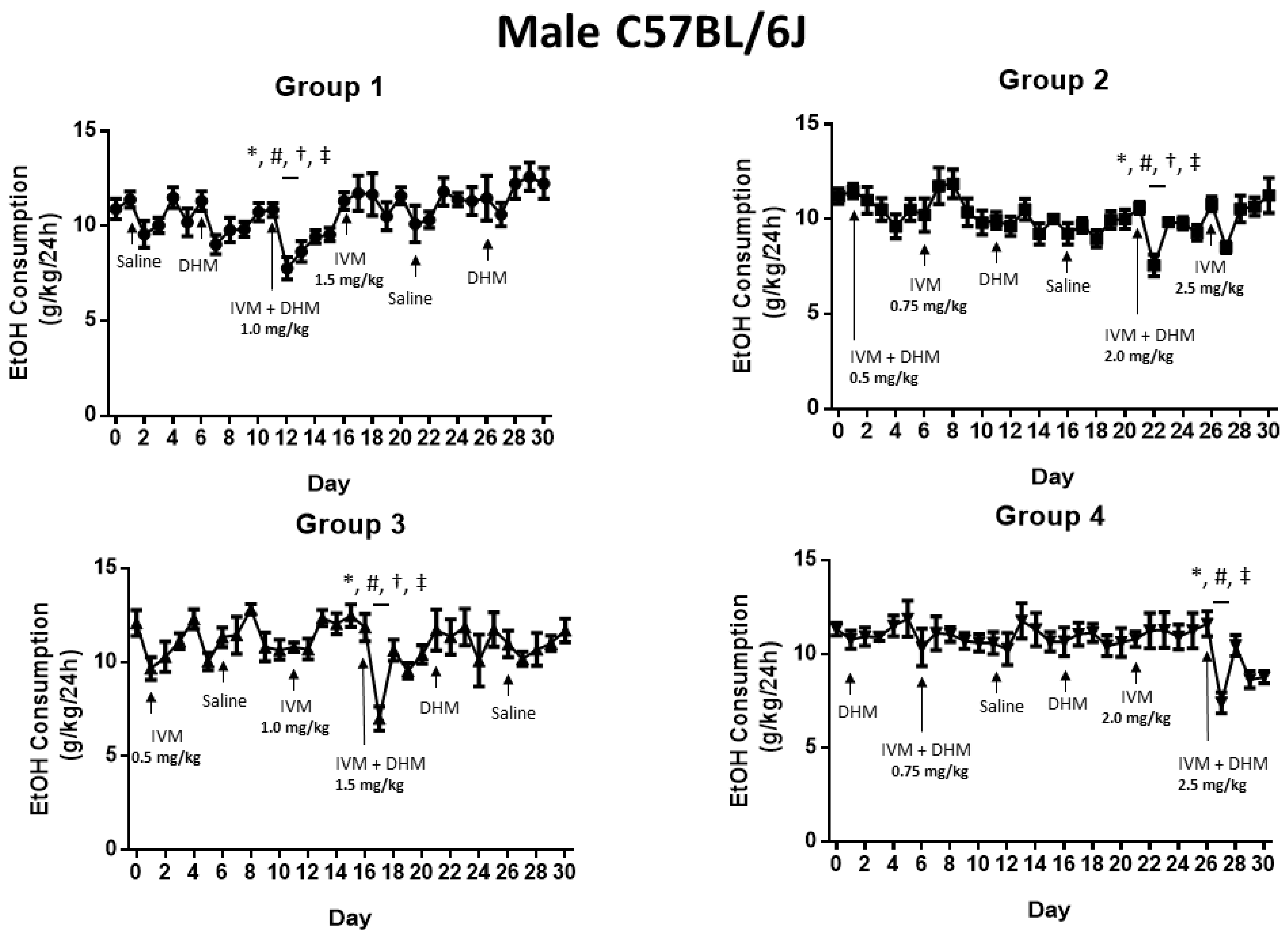
Male C57BL/6J
Group 1
EtOH Consumption (g/kg/24h)
*, #, †, ‡
Saline
DHM
IVM + DHM
1.0 mg/kg
IVM
1.5 mg/kg
Saline
DHM
Day
Group 2
EtOH Consumption (g/kg/24h)
*, #, †, ‡
IVM + DHM
0.5 mg/kg
IVM
0.75 mg/kg
DHM
Saline
IVM + DHM
2.0 mg/kg
IVM
2.5 mg/kg
Day
Group 3
EtOH Consumption (g/kg/24h)
*, #, †, ‡
IVM
0.5 mg/kg
Saline
IVM
1.0 mg/kg
IVM + DHM
1.5 mg/kg
DHM
Saline
Day
Group 4
EtOH Consumption (g/kg/24h)
*, #, ‡
DHM
IVM + DHM
0.75 mg/kg
Saline
DHM
IVM
2.0 mg/kg
IVM + DHM
2.5 mg/kg
Day

B

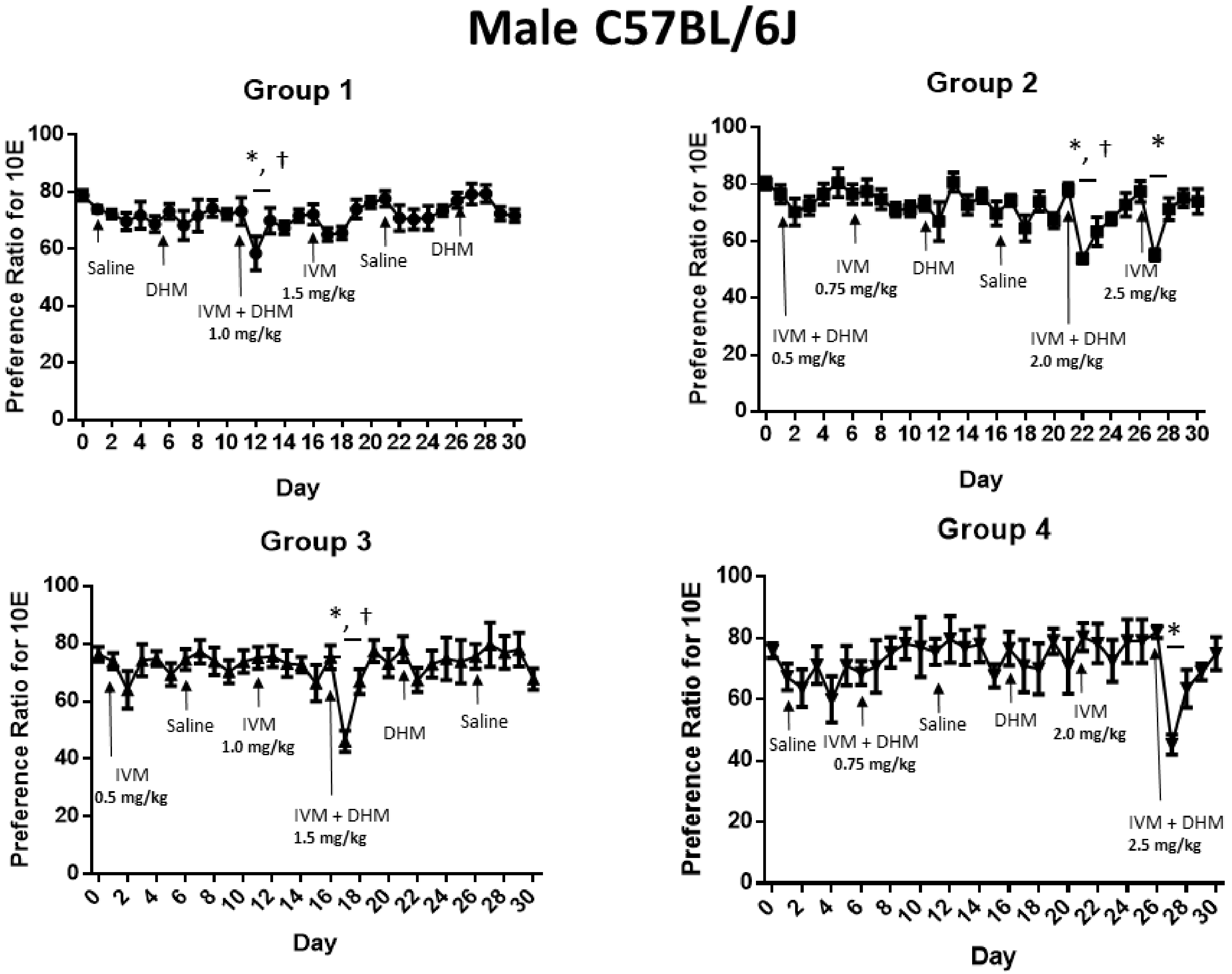

Data S2. IVM, combined with DHM, significantly reduced EtOH intake and 10E preference at lower doses in male C57BL/6J mice. A) IVM (1.0 – 2.5 mg/kg) combined with DHM (10 mg/kg) significantly reduced EtOH intake in comparison to baseline values and IVM controls. B) IVM (1.0 – 2.5 mg/kg) combined with DHM (10 mg/kg) significantly reduced 10E preference in comparison to baseline values within groups and between groups on matched days. The four panels depict the average ± SEM of A) EtOH intake (g/kg/24-h) and B) 10E preference/24-h in male mice randomized into treatment groups. All groups received either IVM dosed at 0.5, 0.75, 1.0, 1.5, 2.0, and 2.5 mg/kg alone or with 10 mg/kg DHM, 10 mg/kg DHM only, or saline i.p. injections. Day 0 is the average of the baseline value over 10 days for that group prior to treatment. Arrows indicate days of injection of the corresponding drug or controls. *$p<0.05$ vs. baseline values, †$p<0.05$ vs. corresponding IVM dose control, #$p<0.05$ vs. DHM control, and ‡$p<0.05$ vs. saline control. N=8/group; RM 2-way ANOVA followed by Bonferonni's multiple comparisons.

A

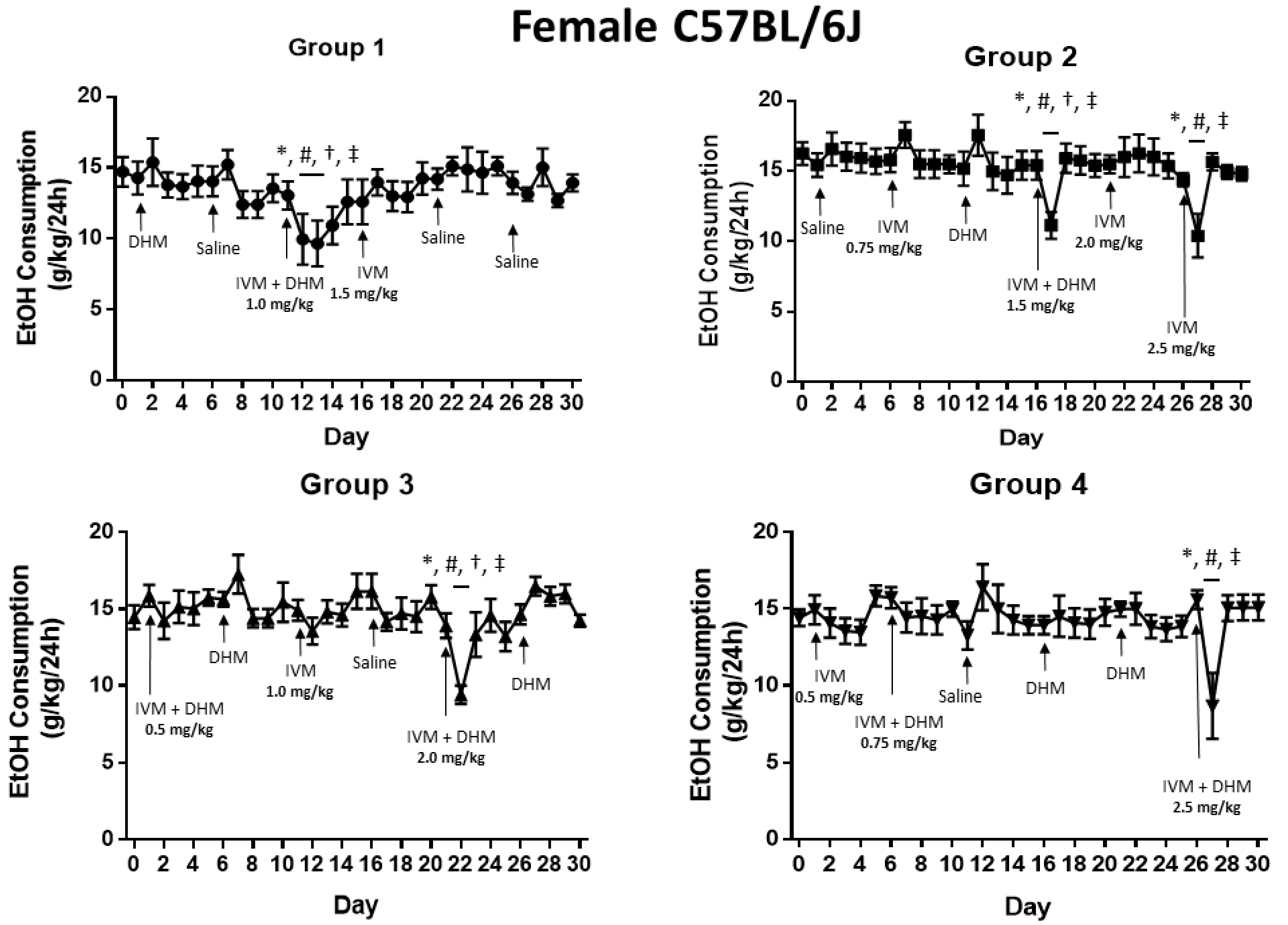
Female C57BL/6J
Group 1
Group 2
Group 3
Group 4
EtOH Consumption (g/kg/24h)
Day
DHM
Saline
IVM + DHM 1.0 mg/kg
IVM 1.5 mg/kg
Saline
Saline
*, #, †, ‡
IVM 0.75 mg/kg
DHM
IVM + DHM 1.5 mg/kg
IVM 2.0 mg/kg
IVM 2.5 mg/kg
*, #, ‡
IVM + DHM 0.5 mg/kg
DHM
IVM 1.0 mg/kg
Saline
IVM + DHM 2.0 mg/kg
DHM
IVM 0.5 mg/kg
IVM + DHM 0.75 mg/kg
Saline
DHM
DHM
IVM + DHM 2.5 mg/kg

B

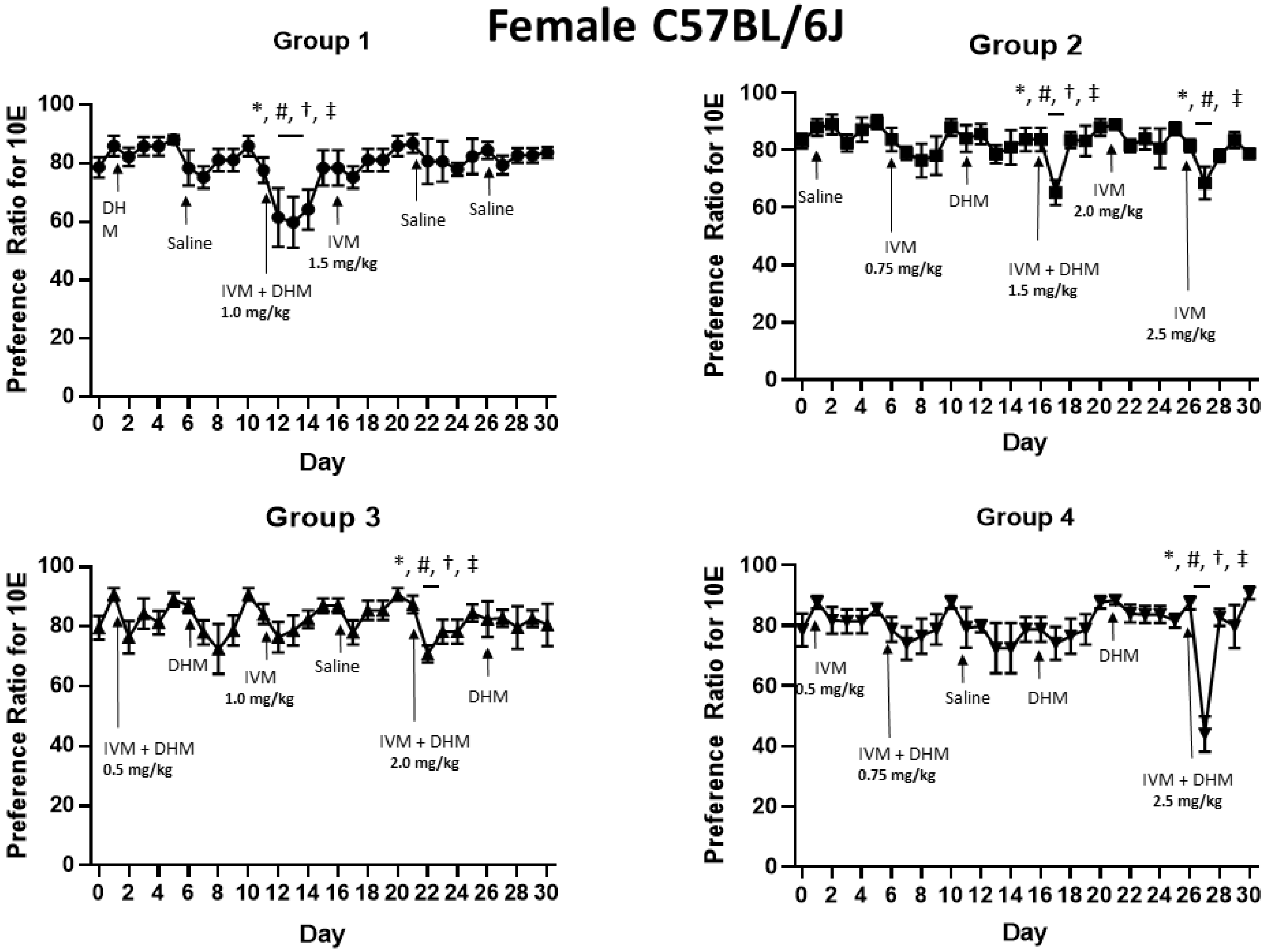

Data S3. IVM, combined with DHM, significantly reduced EtOH intake and 10E preference at lower doses in female C57BL/6J mice. A) IVM (1.0 – 2.5 mg/kg) combined with DHM (10 mg/kg) significantly reduced EtOH intake in comparison to baseline values and IVM controls. B) IVM (1.0 – 2.5 mg/kg) combined with DHM (10 mg/kg) significantly reduced 10E preference in comparison to baseline values within groups and between groups on matched days. The four panels depict the average ± SEM of A) EtOH intake (g/kg/24-h) and B) 10E preference/24-h in female mice randomized into treatment groups. All groups received either IVM dosed at 0.5, 0.75, 1.0, 1.5, 2.0, and 2.5 mg/kg alone or with 10 mg/kg DHM, 10 mg/kg DHM only, or saline i.p. injections. Day 0 is the average of the baseline value over 10 days for that group prior to treatment. Arrows indicate days of injection of the corresponding drug or controls. *$p<0.05$ vs. baseline values, †$p<0.05$ vs. corresponding IVM dose control, #$p<0.05$ vs. DHM control, and ‡$p<0.05$ vs. saline control. N=8/group; RM 2-way ANOVA followed by Bonferonni's multiple comparisons.

A

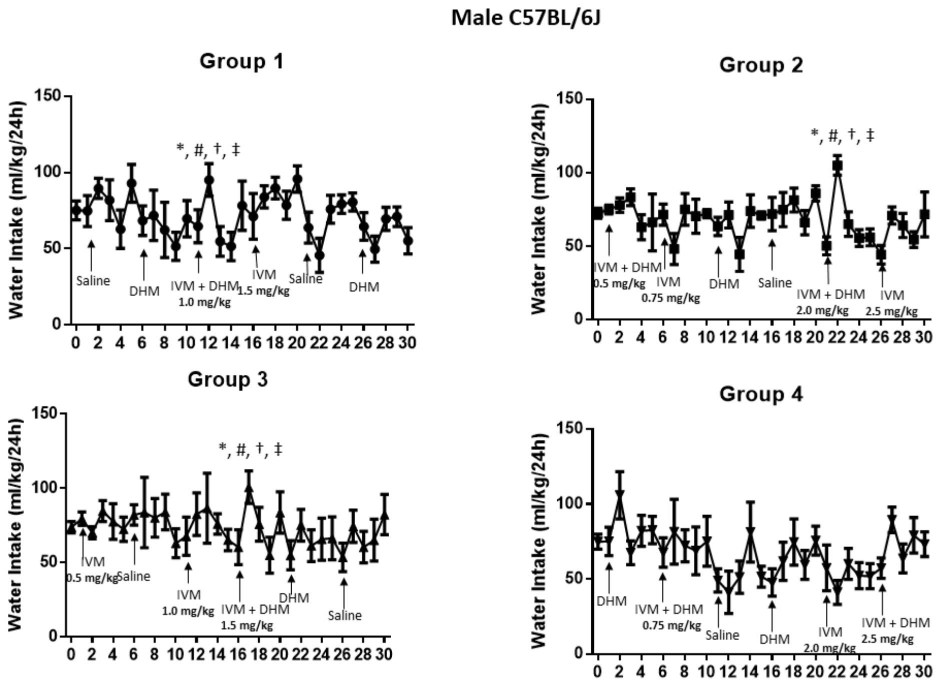

B

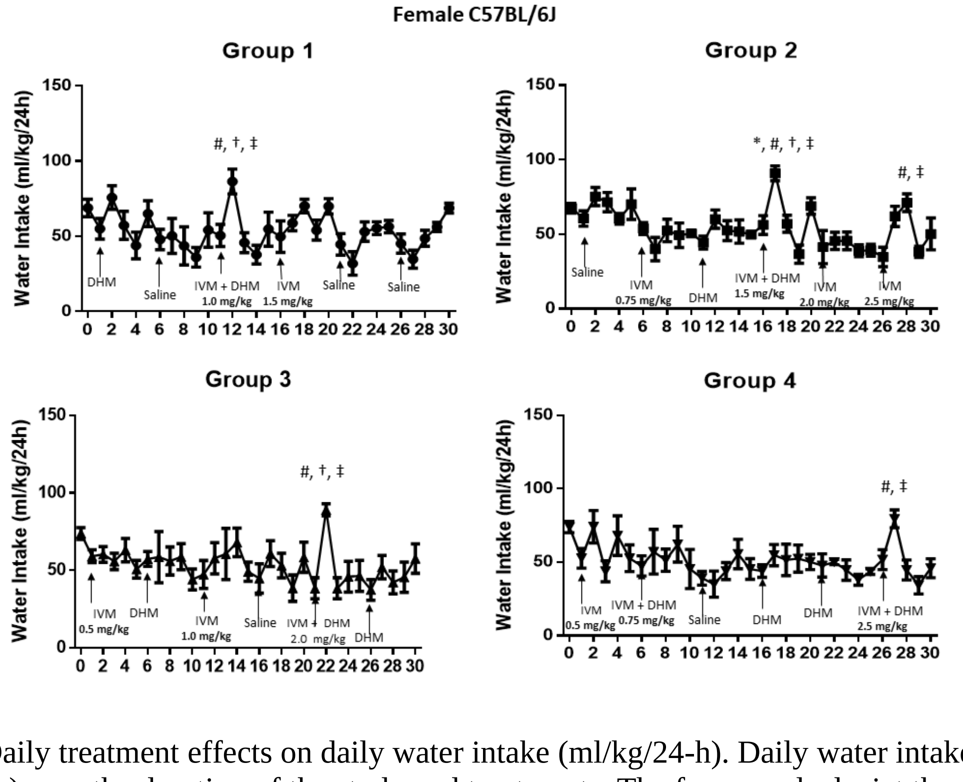

Data S4. Daily treatment effects on daily water intake (ml/kg/24-h). Daily water intake (ml/kg/24-h) over the duration of the study and treatments. The four panels depict the average ± SEM of A) Male water intake (ml/kg/24-h) and B) Female daily water intake (ml/kg/24-h). Day 0 is the average of the baseline water-intake values over 10 days for that group prior to treatment. Arrows indicate days of injection of the corresponding drug or controls. *p<0.05 vs. baseline

values, †p<0.05 vs. corresponding IVM dose control, #p<0.05 vs. DHM control, and ‡p<0.05 vs. saline control. N=8/group; RM 2-way ANOVA followed by Bonferonni’s multiple comparisons.

A

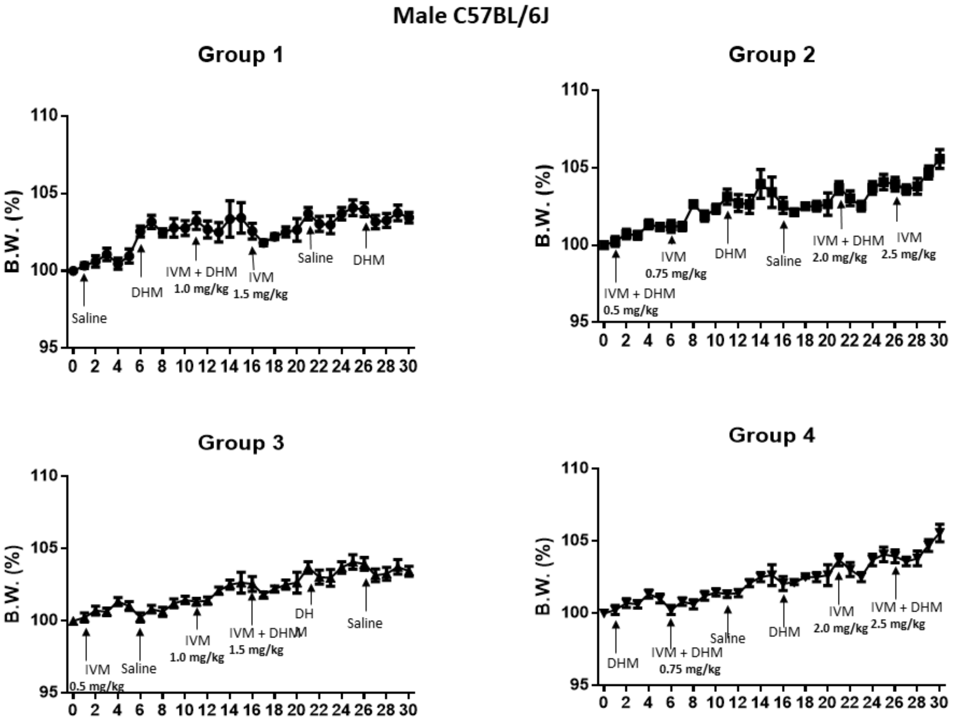

B

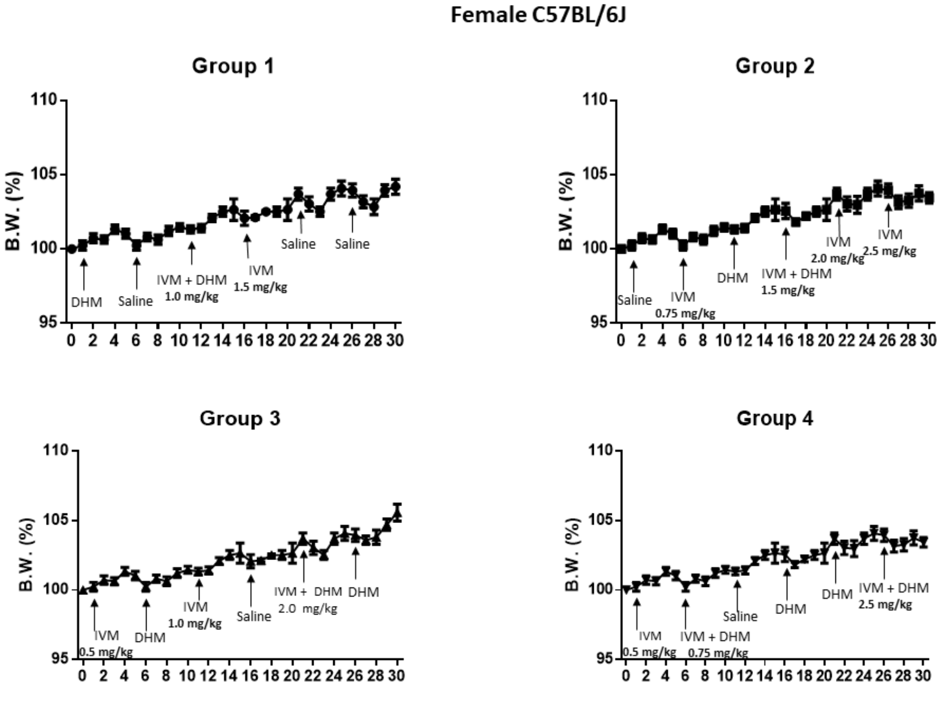

Data S5. Daily treatment effects on body weight (B.W.; %). B.W. % changes over the duration of the study and treatments. The four panels depict the average ± SEM of A) Males and B) Female B.W. % changes. Day 0 is the starting weight average for that group over the 10 days prior to treatment. Arrows indicate days of injection of the corresponding drug or controls. No significance was observed between treatments.

A

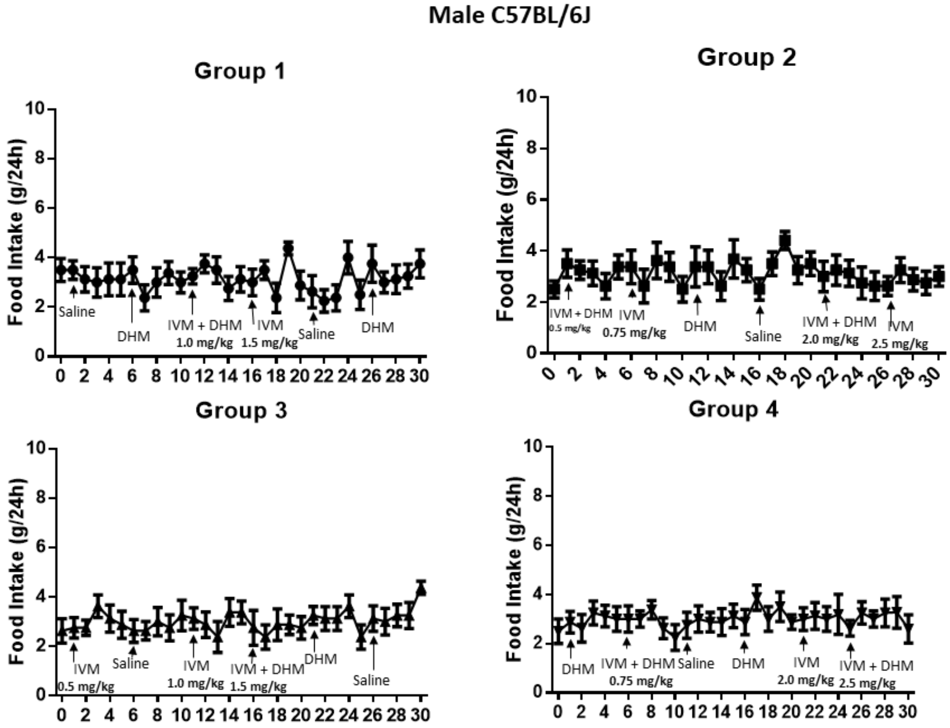

B

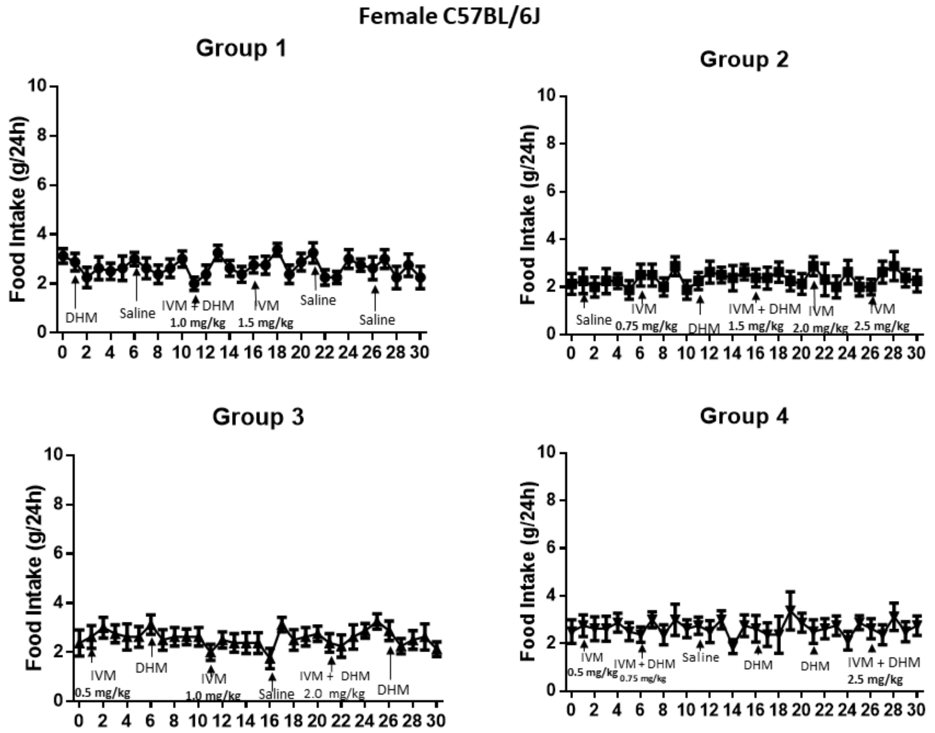

Data S6. Daily treatment effects on daily food intake (g). A) Daily food intake (g) over the duration of the study and treatments. The four panels depict the average ± SEM of A) males and B) Female daily food intake. Arrows indicate days of injection of the corresponding drug or controls. No significance was observed between treatments.
